# A classroom SNS to promote reflective activity in programming learning for children

**DOI:** 10.1186/s41039-018-0069-9

**Published:** 2018-04-10

**Authors:** Kumpei Tsutsui, Hideyuki Takada

**Affiliations:** Faculty of Information Science and Engineering, Ritsumeikan University, 1-1-1 Noji-Higashi, Kusatsu, Shiga, 525-8577 Japan

**Keywords:** Programming education, Reflection, Social networking service, Collaborative learning, Knowledge sharing

## Abstract

In recent years, workshops involving programming on computers have been held in elementary education to promote creative thinking. In our experience of conducting programming workshops for children for over 10 years, we cannot have enough time to let them share and reflect on their creative activity, although reflection is one of the important factors for successful experience-based learning. In order to solve this problem, we have developed a classroom SNS (social networking service) system for children to upload a post and share their project. On the SNS site, they can upload three kinds of contents: action view, code view, and comments. We conducted an experiment to examine if children have performed the sharing and reflection activity with the SNS system during a programming workshop. The result shows that the SNS system would promote motivation for children to reflect on their project.

## Introduction

Programming education is becoming an important element of elementary schools in Japan (MEXT 2011). The government has decided during the Industrial Competitiveness Conference to include programming education in compulsory courses from 2020. The purpose is to gain not only programming skills but also logical thinking skills. As a result, elementary school students have many opportunities to do programming through trial programming classes at a school or workshops.

In experience-based learning, it is very important to reflect on the activities. By doing so, your experience becomes your knowledge, and you can learn from your experience (Kolb 2014). It is essential to be evaluated by others in addition to engaging actively during a class, especially, in programming education (Monroy-Hernández 2007). Scratch (Resnick et al. 2009) which was specially developed for primary school students offers not only an easy programming function but also a function to share and publish the finished project to the world. However, current programming education focuses too much on writing a program itself. Therefore, users do not have much opportunities to present the finished project for evaluation and reflect on their experiences.

In this research, we propose a classroom SNS (social networking service) which enables users to share their project-in-progress as well as their finished project. Children can save their project progress which is useful to reflect on their activities. In addition, the SNS enables children to share knowledge among the classmates. Hence, we assume that the skill disparity will be smaller and creative motivation will increase.

The structure of this paper is as follows: First, we explain the contents of current programming education at primary schools. Then, we emphasise reflection activities focusing on programming education and propose a classroom SNS which aims to promote reflection activities. The result of the experimental implementation of the proposed system is also shown followed by a summary of the research.

### Programming education in elementary school

#### The purpose of programming education

Many attempts have been made to introduce programming to children (Kelleher and Pausch 2005).

The purpose of recent programming education is not to develop human resources who can write a program using a programming language but to gain ‘computational thinking skills’ (Papert 1980; Wing 2006), which would play an important role to learn science (Basu et al. 2016) and build an ability to think logically. To reach an expected result, it is required to think logically on what to do and how to improve just like when using a computer. Another aim of programming education is to understand how computers are used in our daily lives and gain an ability to apply computers in one’s own life.

The primary objective of programming education is not to learn programming language itself but to be able to use programming skills as a tool.

#### Education method

As the interest in programming education increases, programming workshops are held by various organisers and some elementary schools offer pilot programming classes. Programming education in elementary and junior high schools does not have a long history. Therefore, programming language used in workshops and teaching methods vary depending on organisers and teachers. The most frequently used language is Scratch which was specially developed for beginners. According to the research conducted in 2015 by the Ministry of Internal Affairs and Communications in Japan, 36% of organisers use Scratch in programming workshops for elementary school students.

In general, the teaching method can be divided into two types: lecture-style learning and material-based learning.

- Lecture-style learning
  While listening to an instructor’s explanation like a school class, participants learn programming skills with the instructor. To support the participants who cannot keep up with the progress of a lesson, organisers tend to appoint a supporter(s). In most cases, participants are given the time to brush up their finished project when the trainer’s explanation is over. At the end of the lesson, participants are given the time to present their finished project in front of all the members or run the project in a small group. Since this learning type enables organisers to teach many people at once, lectures at elementary schools and one-time workshops are often instructed in this form.
- Material-based learning
  In this learning, organisers distribute teaching materials to participants and respond to questions as necessary. Organisers typically appoint one supporter per four or five children. When the participants complete all the materials, supporters review the contents. Like the lecture-style learning, participants are given the time to brush up their finished project.

The organisers can prepare the materials which match each participant’s skill level. Therefore, a group of children with different programming skill levels can be taught in the same workshop. However, since not all the participants are using the same material in this style of learning, it is not possible to refer to the other participants’ project and teach each other.

#### Challenges of programming education

Most of 1-day workshops emphasise just experiencing programming, resulting in a rare chance to evaluate whether participants acquired the programming knowledge or not. In order to foster computational thinking skills, children are required to have established knowledge and skill for programming as a basis for creating their projects during the workshop. However, it is difficult to measure whether participants have acquired such knowledge, because conducting a paper-based test like a conventional subject does not fit well to the evaluation of the knowledge and skill of programming.

Programming education through continuous learning is still under the development stage in terms of knowledge establishment. Our challenge is to build a computer-based environment for children to establish knowledge at a programming workshop by generating reflection activities.

### Reflection activities in programming education

#### Reflection activities in education

A reflection activity is performed in order for a learner to review the learned content and to establish the gained knowledge. Items related to reflection activities have been added to the current course of study guidance which had been implemented since FY 2010 in Japan. Hence, reflection activities are paid much attention in the last several years.

The value of experiencing will increase by reflecting on the experiences repeatedly over time and thinking about the meanings of experiences, especially in experience-based learning such as programming education. John Dewey said, ‘We do not learn from experience itself. We learn from reflecting on experience.’ In fact, during a class of ‘integrated studies’ at elementary schools, children often create a portfolio which supports them to reflect on their learning activities.

E-portfolio, an electronic version of portfolio, is a collection of students’ work and can be used as a tool to support reflective learning processes such as problem-based learning (Stefani et al. 2007). An application called ‘LoiLo’ allows children to be able to easily create a portfolio from paper notes which they are using daily and share the created portfolio with classmates.

#### Creative thinking spiral

**Fig. 1:**
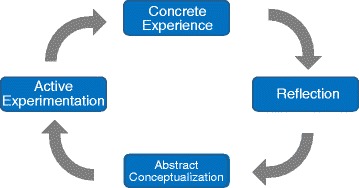
Kolb’s experimental learning model

Kolb has proposed an experience-based learning model as a learning process of experience-based learning. As shown in Fig. 1, this model enables learners to learn from experiences by repeating the following four processes: ‘active experimentation’, ‘concrete experience’, ‘reflective observation’, and ‘abstract conceptualisation (Kolb 2014)’. This model is widely used in school education and in-house training.

In programming education, there is a model called ‘creative thinking spiral’ as a process model to foster creative thinking skills to come up with innovative solutions to the unexpected situations that will continuously arise in people’s lives (Resnick 2007).

**Fig. 2:**
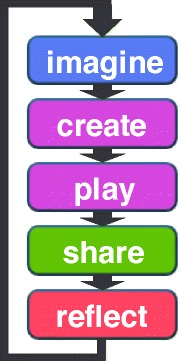
Creative thinking development model

As shown in Fig. 2, this model aims to build creative thinking skills by repeating the processes of *imagine*, *create*, *play*, *share*, and *reflect*. The three processes of ‘imagine’, ‘create’, and ‘play’ are performed during the production stage. On the other hand, ‘share’ and ‘reflect’ are performed by presenting the produced project to others after completing the production.

The difference between Kolb’s experience-based learning model and Resnick’s model is that Resnick’s model includes a sharing process. The sharing process which enables users to reuse the program written by other people is very convenient due to the nature of programming activities (Monroy-Hernández and Resnick 2008). It also aims to nurture the idea of learning from each other based on the ideas and projects of other people by making the project public to others (Monroy-Hernández 2007; Resnick 2007). By sharing the finished project, users can receive feedback which contributes to reflection processes.

#### Challenges of creative thinking spiral in programming education

Scratch is a programming environment developed based on the above-mentioned creative thinking development spiral. Projects developed with Scratch will be published on the web and shared to the world by pressing a ‘share’ button. By sharing with others, other users can give comments on the shared project or improve the project.

**Fig. 3:**
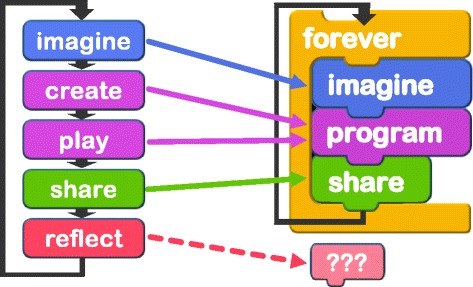
Creative thinking spiral and slogan of Scratch

Scratch aims to build an online community where users can enhance their creativity while they do programming at home. When programming education becomes more common in the future, it is necessary to enhance creativity not only in an online community but also in a real-world community. However, ‘share’ and ‘reflect’ processes are not easy to be implemented in a real-world workshop. Scratch’s slogan is ‘imagine’, ‘program’, and ‘share’ and does not include ‘reflect’ in comparison to the creative thinking spiral model as can be seen in Fig. 3. As an opportunity to ‘share’ and ‘reflect’, workshop participants may have time to present their project at the end of the workshop. In this case, however, the number of opportunity to ‘share’ and ‘reflect’ is only once. Moreover, since ‘share’ and ‘reflect’ are done at the end of the workshop, there will be a time gap between ‘reflect’ and the next concept building (imagine). In our experience in organising programming workshops, most of the children leave the workshop place without enough reflection on their activity and come back to the next workshop with almost forgetting what they did in the last workshop.

Therefore, we propose a tool that allows the participants to share their projects many times during a workshop and aim to increase the number of opportunities not only to share but also to reflect. We think that posting project-in-progress enables children not only to understand their own project process after completing the project but also to gain some ideas from other children’s posts. Some research proposed systems to share project-in-progress in programming education for elementary school students. In Jots, the users share screenshots of Scratch screen and comments on the shared screen (Rosenbaum 2009). By doing so, it has been reported that there were various advantages such as recognising the usefulness of mistakes and comparing the learning process with others. However, sharing screenshots provides only the appearance of the program but not how the program works. There is also a research which proposed a system to create project progress documents of a workshop for children and share the data on the web (Katterfeldt and Schelhowe 2014). In this research, however, it was found that documenting the project progress is not suitable for reflection as it requires children additional efforts. Tools need to be user-friendly so that the children can easily post and browse as those actions occur many times during a workshop. Therefore, we intend to solve these issues by building a SNS site as its users are not required complicated operation to share, can easily view own posts, and can write comments on others’ posts.

Although using the existing SNS sites such as Facebook group could be a possibility as described in a literature (Wang et al. 2012), we believe that the structure of a post needs to be more well-organised to be used for sharing the project-in-progress in programming workshops; the ‘post-and-commenting’ structure in the existing SNS sites is too general, leading to additional efforts required to express children’s thoughts while working on their project.

## Methods

### Classroom SNS for supporting reflection

#### System overview

In this research, we build a classroom SNS that allows children to post and browse during a workshop in order for them to repeatedly share and reflect on their projects. In programming workshops, a program (organised by *blocks* in Scratch) and how the written program works are the necessary elements to reflect on what they learned. In addition to those two elements, we allow users to post the comments on the project.

Moreover, to generate sharing and reflection many times in one workshop, many posts to the SNS need to be made. Therefore, we enable the children to post with simple operation so that posting will not disturb the workshop progress and make children want to post.

**Fig. 4:**
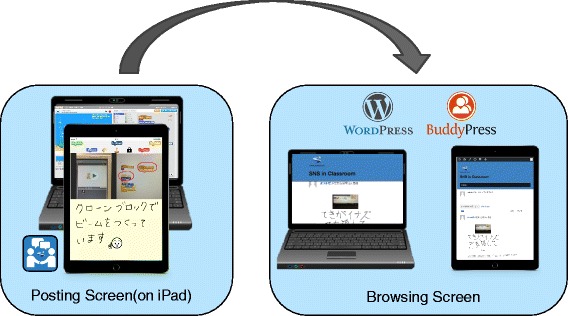
Flow from posting to browsing
  To post to the SNS, we adopt an iPad which is easier for children to operate than a personal computer. The screen of the iPad application is shown in Fig. 5.

As shown in Fig. 4, the system consists of an iPad application for posting and a SNS site for browsing the posted contents. The details of the iPad application and the SNS site are described below.

- iPad application

**Fig. 5:**
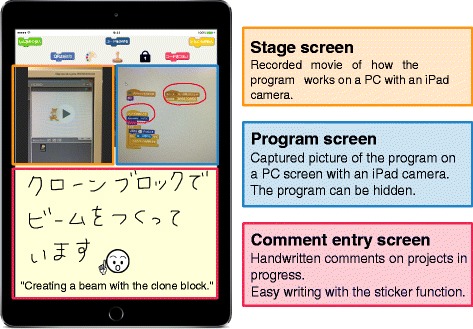
Posting screen on iPad
  Projects in a programming workshop are often created on a personal computer. When sharing the stage screen, users may share by capturing with the ‘print screen’ function. However, in order to reduce the complexity of the operation, we let users capture the stage screen by taking a picture of the screen with an iPad camera. How the program works is recorded as a movie and the program is captured as a still picture. In addition, children can freely write on the captured image of the program by handwriting. As we describe later, the program image can be hidden. The users handwrite the explanation of the program and comments on the comment entry screen.
  The users post these three items (stage screen, programs, and comments) in one screen on the proposed SNS with the application browser. When the posting is completed, the system will notify other users on their browsers.
- SNS site
  Posts are shared on the SNS site. Based on WordPress, the SNS site incorporates with BuddyPress which is a plug-in that creates a community site. Just like a standard SNS, each user can view other people’s posts by logging in with own user ID and password. The proposed SNS site can be accessed regardless of the terminal type. Therefore, the users can browse at home after the workshop is over.

#### Function to post with fun

The system has unique functions to motivate children to post their project-in-progress.

**Fig. 6:**
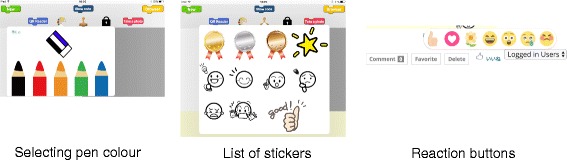
Enjoyable posting function
  In addition to the text entry, the system has a sticker function. Most of messenger applications such as Facebook Messenger offer a sticker function which enables users to easily express their emotion. Examples of stickers are given in Fig. 6. Since children sometimes create a game which is difficult to clear, the stickers include negative emotions.
- ‘Like!’ button
  SNS such as Facebook has a ‘Like!’ button to show viewers’ reactions to posts. We will provide similar reaction buttons in the proposed SNS as shown in Fig. 6. This allows viewers to easily respond to the posts. We think receiving reactions will lead to motivation improvement of the receivers.

- Handwriting and stickers
  Keyboard typing is not easy and takes time for children. Therefore, we adopt handwriting input for text entry. It saves time and offers enjoyable time. Moreover, users can select the font colour to easily emphasise the comment as shown in Fig. 6.

#### Non-display function of posted images

In the posting screen of the iPad application shown in Fig. 5, users can post without showing a captured image of the program. This function is added with an intention that requiring one additional operation will activate actual interaction among children such as teaching each other. If the image of the program is hidden, other participants can only view how the program works and posted comments but not what kind of blocks are used in the program. If they wish to see the image of the program, they need to ask directly to the person who posted the image.

**Fig. 7:**
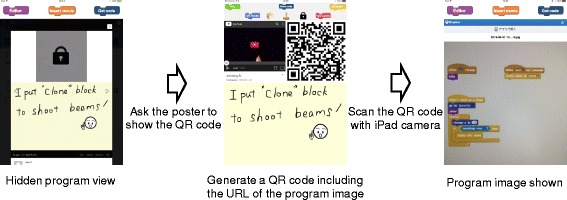
Non-display function of posted image

Figure 7 shows how the non-display function of posted image can be used. The poster can choose not to show the program image when he/she posts. When the poster is asked to show the image, he/she presses the ‘show code’ button. All captured images are stored on a cloud file storage and when the ‘show code’ button is pressed, a thumbnail of all images will be displayed. When the target image is selected from the thumbnail, a QR code including the image address will be displayed on the iPad. Participants who wish to view the program can access the image of the program saved on the cloud storage by reading the QR code with own iPad.

#### Generating creative thinking spiral with the proposed system

**Fig. 8:**
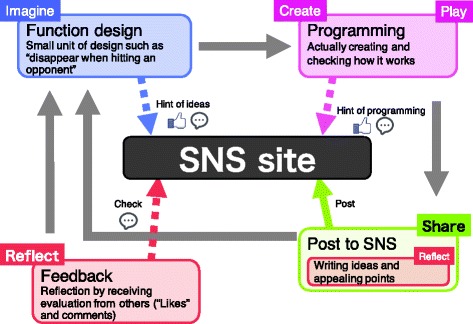
Creative thinking model on the proposed system
  Imagination happens when you design a new function. This design step is not for a whole project design but for a small unit design such as what to make and what kind of function to add. If you do not know what to make or not come up with an idea, access the proposed SNS and grab some ideas from others’ posts. During viewing others’ posts, you can press the ‘Like!’ button and write a comment.
- Create and play
  Once you decide what to make, do programming following the plan. If you do not know how to do programming, access the SNS and get some hints on coding from others’ posts. When viewing others’ posts, you can press the ‘Like!’ button and write a comment. Once programming is done, run the project and check how it works.
- Share
  When creating a certain function is completed, a user posts a movie of the stage screen, a program capturing image, and a comment on the proposed SNS. Using an iPad camera, the user takes a picture of the part you want to share with other participants and the part you want to record for reflection. In addition, the user enters comments against the program of project-in-progress. Once the posting is done, it will be notified to other participants’ PCs.
- Reflect
  There are two opportunities to reflect. First opportunity is when a user posts on the SNS. The user writes a comment against the project-in-progress. When writing a comment, he/she reflects by looking back where he/she put a lot of thoughts and how he/she created the project. Second opportunity is when the user receives ‘Like!’ or comments from other participants on their posts. We think that users can reflect objectively by receiving evaluation from others.

We aim to motivate children to repeat reflection by generating ‘creative thinking spiral’ many times during a workshop using the proposed system. This section and Fig. 8 explain when each process occurs in a workshop.

- Imagine

## Results

### Applying the proposed SNS to programming education

#### Application procedure

In order to verify the effectiveness of the proposed system, we conducted experiments at programming workshops organised by NPO Super Science Kids. Table 1 provides the summarised information of the workshops.

**Table 1:** Basic information of the workshops

Date	Age	No. of participants	Contents
		(with experience)	
6th Oct	9–10	5 (5)	Move the robot (Romo) with Scratch (3 h)
6th Nov	8–12	5 (3)	Make shooting game (3 h)
4th Dec	8–12	7 (3)	Make snow playing game (90 min)
		Total 17	

The workshop held on October 6 was an irregular workshop that participants wrote a program with Scratch for an educational robot called Romo. Other workshops were regular programming workshops using Scratch only. There were participants who have experienced Scratch programming in all workshops.

In order to conduct these experiments, we distributed a card with a user ID and a password for logging into the proposed SNS. In addition to a laptop PC for creating a project, we distributed one iPad per participant for posting. Hence, all the participants were able to post at any time during a workshop. Moreover, we let the participants bring back home the card with the log-in information so that they could access the SNS at home.

We installed two cameras to record how the children behave during the workshops. At the end of each workshop, we asked children to fill in a questionnaire regarding the proposed SNS. Table 2 provides the main items of the questionnaire. The questionnaire sheet was given in a pretty format with illustrations so that children could feel easy and motivated to answer. Furthermore, to investigate if the participants repeatedly reflect even after the workshops, we recorded the time of the accesses after the workshop and user IDs of those accesses.

**Table 2:** Main items of the questionnaire

	Question	Answer format
Q1	How did you feel to post your project on SNS using iPad?	Five-point scale
Q2	How did you feel with iPad operation?	Three choices
Q3	When did you post? (2nd and 3rd workshops only)	Three choices
Q4	In what kind of occasions did you access SNS from a PC?	Free writing
Q5	Did you use the function to hide the program?	Two choices
Q6	Please specify the reason of Q5.	Free writing
Q7	Would you like to use the SNS next time?	Two choices
Q8	Please specify the reason of Q7.	Free writing

#### Contents posted to SNS

**Fig. 9:**
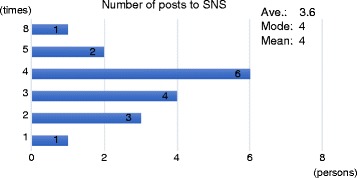
The number of post to SNS

During the workshops, children repeatedly posted the production process of their project. As shown in Fig. 9, the average number of posts per child is 3.7 times while the maximum number is 4.

Posts can be divided into two types: useful for self-reflection and useful for knowledge sharing.

**Fig. 10:**
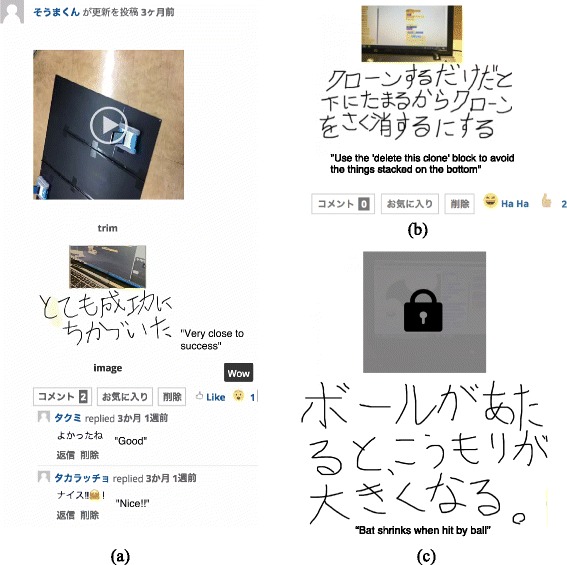
Examples of posts **a** for self-reflection and **b** for knowledge sharing **c** with program screen hidden

The former type of posts includes only impressions and thoughts which can be understood by only the poster. They do not include how the program works. Such examples are given in Fig. 10a. In this example, the comment says, ‘very close to success’ which is written for himself. Those posts/comments are useful for recording the production process and reflecting by the poster himself. Moreover, as the video was posted together to show how the project works, other participants gave comments and reactions as seen in the lower part of Fig. 10a.

On the other hand, the latter type of posts is useful for knowledge sharing although they are primarily posted for self-reflection. Since those posts describe how the program works and how the poster wrote the program, they are useful for other participants. Examples are given in Fig. 10b. In this example, the comment describes an issue of the program and its solution.

Some posts were submitted without showing program image using the non-display function as seen in Fig. 10c. Some of them were useful for both knowledge sharing and poster’s reflection. Table 3 shows the results of the questionnaire on the non-display function. While nearly half of the participants answered that they have posted using the non-display function, only four participants have viewed hidden program screens.

**Table 3:** Questions on the non-display function

Question	Answer	Votes
Did you use the non-display function?	Yes	9
	No	8
Did you view the hidden program?	Yes	4
	No	13

#### Children behaviour during workshops

This section describes how the children behaved when they were posting to the SNS during the workshops.

**Fig. 11:**
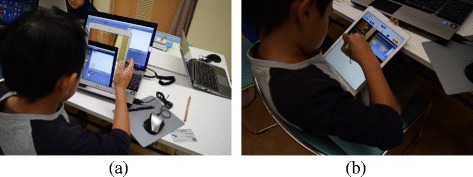
Children behaviour. **a** Capturing screen. **b** Writing a post

Table 4 shows the result of the questionnaire on usability of the SNS. The operation of iPad has mainly two aspects: take a picture of the PC screen as shown in Fig. 11a and write the contents of a post as shown in Fig. 11b. For a question about iPad operation, eight answered ‘easy’ while eight answered ‘difficult’, and the ratio of those who answered difficult was almost half in every workshop. The reason for answering ‘easy’ was mostly ‘because I always use it’. On the other hand, the reasons for choosing ‘difficult’ were such as ‘because I have not used it much’ and ‘because doing programming was difficult’. From these answers, it seems that the operability depends on whether they are used to using iPad or not. However, we did not receive any question during the workshops regarding the SNS posting and did not observe any children who were having problems with the SNS operation. In addition, the average number of posts made by the children who felt difficult was 3.75 while that by those who felt easy was 3.5. Although half of the children felt ‘difficult’ to use the iPad application, it seemed not to affect the number of posts to the SNS. On the other hand, the page navigation in the application is expected to be improved because two of the children raised the difficulty of properly choosing the function to use.

**Table 4:** Questions on usability of the SNS

Question	Answer	Votes
Was it easy to operate iPad?	Easy	8
	Fair	1
	Difficult	8
Was it fun to post to SNS your project using iPad?	Very fun	8
	Fun	4
	Fair	5
	Not so fun	0
	Not fun	0

Responses to the question asking whether it was fun to post to the SNS were mostly positive. The reasons of choosing ‘fun’ were because participants enjoyed sharing the posts. Examples of responses were ‘I like my posts being viewed by everyone’ and ‘I can get a hint from others’ posts’. In addition, operability was also given as a reason of choosing ‘fun’. Examples of answers were ‘it was fun to hide the program’ and ‘because I can write a message’. Some of the reasons for selecting ‘fair’ were ‘because I did not have much time to use it’ and ‘I did not understand how to use’. However, as there was no relation between the operability of iPad and the answer to whether it was fun to post to the SNS or not, we assume that operability did not affect the pleasure of posting to the SNS.

Table 5 shows the results of the questionnaire on when children used the SNS. As the timing to post, the most frequent answer was ‘when I come to a break’. Children often stopped their task at the timing that they thought it was a good moment to post. The most frequent answer for when to view the posts was when the children wanted to refer others’ idea. There were actually some children who posted ‘let’s view the SNS because I cannot understand’ before viewing others’ posts. From these facts, we could conclude that children used the SNS according to their own pace.

**Table 5:** Questions on when to use the SNS

Question	Choices	Votes
When did you post to SNS?	When I build a new function and want to be viewed by others.	3
(multiple answer allowed)	When I come to a break.	8
	When I want to store to reflect later.	3
When did you view the SNS?	To refer others’ idea.	11
(multiple answer allowed)	To confirm if my record is successfully posted or not.	6
	To see other’s progress.	1
	Not viewed.	2

**Fig. 12:**
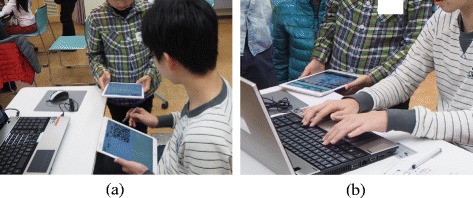
Communication among children promoted by the non-display function. **a** Showing the hidden program. **b** Sharing the knowledge

We observed some children asked the poster about the hidden program. Figure 12a is a picture of when a child is reading a QR code on the poster’s iPad to view the hidden program. It took some time to read due to a light reflection or operation mistakes. Although those two children met for the first time at the workshop, they cooperated and had a communication. Even after the image was displayed, the poster run the project to show how it works and explained other functions as seen in Fig. 12b. They not only communicated in SNS but also had an actual conversation. Regarding the reason why some of them used non-display function was because they simply wanted to use without any particular reason. On the other hand, some of them used the function with an intention to hide the program in order not to show much of the contents or everything. It is obvious that they did not use the non-display function to activate actual communication among them. However, we think that the number of opportunities for them to communicate was increased because of the non-display function.

#### SNS usage after the workshop

**Fig. 13:**
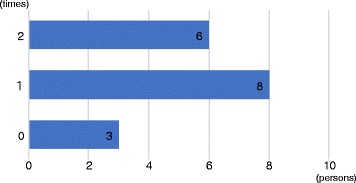
The number of accesses to the SNS after the completion of workshops

We have investigated the number of accesses to the SNS and contents of the posts after completing each workshop. Figure 13 summarises the number of accesses after the workshops. It turns out that many children accessed the SNS even after workshop was finished. In addition, there were four posts. One of them was a post of a game developed at home. Many comments and reactions were given to the post; therefore, we can say that the communication continued even after the workshops ended.

## Discussion

The purpose of this research is to promote reflection activities by enabling workshop participants to post their project-in-progress to the SNS. Here, we discuss whether the SNS actually promoted reflection and whether SNS is suitable as a tool to promote reflection. We also evaluate the effectiveness of the proposed SNS.

### Promoting reflection activities

All the workshop participants posted at least once during the workshop. It means that at least one reflection activity per participant occurred during the workshop. Many children posted several times and most of those posts were made when they came to a break; therefore, reflection occurred many times during one workshop. In addition, as there were accesses to the SNS even after the workshops, those participants had opportunities to review their project once again and had reflection activities.

In order to investigate if the participants want to keep using the SNS, we included questions shown in Table 6. In response to the question whether they want to use the SNS in the next workshop, 12 of them answered ‘Yes’ and 2 answered ‘No’. There were more children who chose ‘because it was easy to understand how I created the program’ than those who chose ‘because I can see how other participants created a program’. The reason of choosing the former option could be because others’ posts were useful for reflection while the reason of choosing the latter option might be because others’ posts were useful for knowledge sharing. From the result of this questionnaire, children seemed to be feeling that the production process was easy to understand with using the SNS.

**Table 6:** Questions on continuous usage of the SNS

Question	Choices	Votes
Do you want to use the SNS	Yes	12
in next workshop?	No	2
	No answer	3
Why do you want to use it again?	Because it was easy to understand	5
(multiple answer allowed)	how I created the program.	
	Because I can see how other participants	3
	created a program.	
	Both	4

**Fig. 14:**
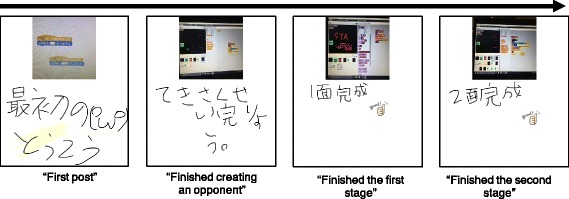
Transition of post content

Moreover, there were some posts which follow production process until the completion as shown in Fig. 14. Such posts clarified the production process and are useful to reflect on the whole workshop process.

From the given facts above, we can conclude that the proposed SNS could promote the reflection activities.

### Using a SNS as a tool to promote reflection activities

Recording the production process by posting to the SNS resulted in receiving comments and ‘Like!’ reactions even if posts were for posters’ own interests. We think that receiving reactions from other participants leads motivation improvement. It is an advantage of the SNS that allows users to easily react to others’ posts.

Recording the production process should not decrease the time for creating a project. In that respect, the SNS has an advantage that it is easy to post compared to other blog and web services. Most of the children who attended the workshops did not face any difficulties in operating the proposed SNS and enjoyed using it. Therefore, they enjoyed posting the processes without spending too much time.

Moreover, displaying others’ posts and own posts on the same page in the timeline format led users to view others’ posts. Since writing a program in a regular workshop is often an individual task, participants do not have a chance to speak with other participants or to know what kind of program other participants are writing. From the questionnaire conducted, we assume that the proposed SNS provides users a way to know what others are doing. In addition, we think that the proposed SNS improved users’ motivation by putting the users being seen by other participants.

From the reasons mentioned above, we think that the proposed SNS promotes reflection activities.

### Discussion on the validity of the proposed SNS

Two children responded negatively to the question asking if they want to use the SNS in a next workshop. They have participated in several workshops before our experimental workshops. As they have not recorded their production processes in the previous workshops, we think they could not see any advantages in making posts to the SNS. Moreover, we assume that they could not learn much from beginners’ posts from a knowledge sharing perspective.

From these facts, the proposed SNS is effective for beginners at present. In order to make this SNS effective for experienced participants, the posts with high-level contents are necessary. Hence, it is important to collect a large number of posts continuously during workshops and build the SNS as a knowledge centre.

## Conclusion

We have presented a classroom SNS for promoting reflection activities and our experience of its application in actual programming workshops. Our contribution is that we have shown what functions are required to promote the reflection activities in programming workshops and revealed their effectiveness by applying the SNS to actual workshops and conducting the analysis with the questionnaire and the observation of children’s behaviour.

In programming workshops, participants cannot spend much time for reflection which is an important process of experience-based learning. Therefore, we have proposed a SNS where users post production processes aiming to promote reflection and knowledge sharing among the participants.

As a result of applying the proposed SNS to actual workshops, we have found that the proposed SNS provided many opportunities for children to post their production processes and reflect on their activities. Therefore, we have concluded that the posts are useful to reflect on their own experiences. In addition, we have confirmed that others’ posts are useful from a knowledge sharing perspective. However, knowledge sharing sometimes did not happen due to the skill level of participants and contents of posts.

This research still has the limitation in terms of evaluating the learning performance in programming workshops. Although the reflection which has been triggered by using this SNS site might change the children’s thinking process, it is not clear yet how the system improves the learning activity in quantitative measures such as learning time and the volume of created programs. Our future work includes applying this system to more actual workshops to collect more posts and verifying if children could accumulate and establish the knowledge from reflection activities.
